# 303 Burn Rehabilitation Training for the Pediatric Burn Critical Care RN

**DOI:** 10.1093/jbcr/irad045.278

**Published:** 2023-08-29

**Authors:** Robyn Bartlett, Trang Bui, David G Greenhalgh, Debbie Minter, Tina Palmieri, Kathleen S Romanowski, Soman Sen

**Affiliations:** Shriners Children’s Northern California, Sacramento, California; Shriners Children’s Northern California, Sacramento, California; University of California, Davis and Shriners Hospital for Children, Northern California, Sacramento, California; Shriners Children’s Northern California, Carmichael, California; UC Davis and Shriners Children’s Northern California, Sacramento, California; Shriners Hospital for Children Northern California, Sacramento, California; Shriners Children’s Northern California, Sacramento, California

## Abstract

**Introduction:**

New registered nurses (RNs) in the burn pediatric intensive care unit (PICU) need extensive pediatric, critical care, and wound care training. Each of these specialties present a challenging learning curve to the new RN and can overshadow the RN’s focus on burn rehabilitation. Our goal was to improve the RN’s understanding of the importance of early rehabilitative care and their role in the provision of burn rehabilitation in the burn PICU setting.

**Methods:**

We developed an interdisciplinary program which allowed the RN to work alongside burn physical and occupational therapists. The aim of the program was to teach the participants the importance of early and consistent splinting, positioning, mobilization, and other therapeutic interventions and to learn how to incorporate these techniques into their nursing plan of care. Over two years, nine RNs participated in a four-hour shadow program with a senior physical and occupational therapist. The therapists provided direct hands-on training with patients across the care continuum focusing on competencies aligned with the American Burn Association’s Burn Nurse Competencies. The RNs completed a self-assessment tool to score their self-perceived knowledge related to burn rehabilitation. The self-assessment included a 10-question Likert-scale survey, based on Benner’s Novice to Expert Model. Scoring aligned with the Benner Stages of Clinical Competence: 10 = Novice, 20 = Advanced Beginner, 30 = Competent, 40 = Proficient, and 50 = Expert. The survey was completed pre-intervention, immediately post-intervention, and 3-6 months post-intervention.

**Results:**

Nine RNs completed the serial surveys. The median years of nursing experience was 2.5 (2-3.5) years. The training was provided at a median time of 4 (3.1-4.6) months after orientation completion. The median score for the pre-intervention assessment was 19 (16-25). The immediate post-intervention score significantly increased to a median of 30 (25-31) (p=0.006) and was maintained at 3-6 months post-intervention (32 (29-37) p=0.0002). There was no significant difference in assessment scores between the immediate post intervention and the 3-6 month post intervention assessment (p=0.25). All median values are median (interquartile range) and significance level was set at a p< 0.05.

**Conclusions:**

New RNs in the burn pediatric critical care unit have a tremendous learning curve. Our training model demonstrates that there may be improved competency when learning is introduced in staged approaches. Additionally, burn rehabilitation in the PICU requires a transdisciplinary understanding of the patient’s needs. Our training program demonstrates that collaboration across professional disciplines to provide nursing education can improve the clinical competency of new RNs.

**Applicability of Research to Practice:**

Identifying more efficient and effective methods of new RN training can improve the delivery of nursing care and improve patient outcomes.

